# Supplementary material for: Exploring Codon Adjustment Strategies towards Escherichia coli-Based Production of Viral Proteins Encoded by HTH1, a Novel Prophage of the Marine Bacterium Hypnocyclicus thermotrophus
Source: Viruses. 2021 Jun 23;13(7):1215. doi: 10.3390/v13071215 (PMC8310279; doi:10.3390/v13071215)
Supplement: Supplementary file 1 [file viruses-13-01215-s001.zip › Supplementary Data_Arsin_et_al_MDPI_MRevised.pdf]

Supplementary Table 1: The list of prophage-associated CDs identified in the *Hypnocyclicus thermotrophus* genome and their putative functions, predicted by NCBI and PHASTER annotation pipelines. GenBank accession numbers represent the corresponding proteins from each locus, pseudogene entries are noted instead of an accession number. E-values correspond to PHASTER annotation of each entry. Non-available E-Values marked as "NA'". Entry marked with an asterisk (*) is not found in the three defined regions as it was not annotated by PHASTER (ND), but was manually curated.

Region	GenBank	Locus tag	NCBI annotation	PHASTER annotation	E-Value
1	WP_134112568.1	EV215_RS03310	phosphoribosylformylglycinamidine synthase	putative phosphoribosyl formylglycinamidine (FGAM) synthase II	9.85e-36
1	WP_134112569.1	EV215_RS03315	5-(carboxyamino)imidazole ribonucleotide mutase	5-(carboxyamino)imidazole ribonucleotide mutase;	NA
1	WP_134112570.1	EV215_RS03320	5-(carboxyamino)imidazole ribonucleotide synthase	5-(carboxyamino)imidazole ribonucleotide synthase;	NA
1	WP_134112631.1	EV215_RS03325	phosphoribosylaminoimidazolesuccinocarboxamide synthase	PHAGE_Synech_S_CAM4_NC_031900: hypothetical protein	6.71e-51
1	WP_134112571.1	EV215_RS03330	amidophosphoribosyltransferase	PHAGE_Pseudo_NV1_NC_042107: hypothetical protein	7.44e-09
1	WP_134112572.1	EV215_RS03335	phosphoribosylformylglycinamidine cyclo-ligase	PHAGE_Synech_S_MbCM100_NC_023584: PurM	1.46e-80
1	WP_134112573.1	EV215_RS03340	phosphoribosylglycinamide formyltransferase	PHAGE_Prochl_P_SSM7_NC_015290: PRGA-formyltransferase	3.66e-26
1	WP_134112574.1	EV215_RS03345	bifunctional phosphoribosylaminoimidazolecarboxamide formyltransferase/IMP cyclohydrolase	5-aminoimidazole-4-carboxamide ribonucleotide formyltransferase/IMP cyclohydrolase	5.51e-87
ND	WP_134112775.1	EV215_RS04350	DUF262 domain-containing protein *	-	NA
2	WP_134112776.1	EV215_RS04355	HNH endonuclease	PHAGE_Mycoba_Dlane_NC_042317: baseplate hub assembly protein	4.36e-05
2	WP_134112777.1	EV215_RS04360	hypothetical protein	hypothetical protein	NA
2	WP_134112778.1	EV215_RS04365	recombinase family protein	PHAGE_Lactoc_28201_NC_031013: protector from prophage-induced early lysis	3.17e-33
2	WP_134112779.1	EV215_RS04370	recombinase	PHAGE_Deep_s_D6E_NC_019544: recombinase	1.75e-06
2	WP_134112780.1	EV215_RS04375	recombinase family protein	PHAGE_Bacill_Gamma_NC_007458: site-specific recombinase	1.36e-34
2	WP_134112781.1	EV215_RS04380	hypothetical protein	hypothetical protein	NA
2	WP_134112782.1	EV215_RS04385	hypothetical protein	PHAGE_Clostr_phiCT453A_NC_028991: exonuclease A	0.0
2	WP_134112783.1	EV215_RS04390	DUF2815 family protein	PHAGE_Clostr_phiCT453A_NC_028991: hypothetical protein	7.62e-68
2	WP_134112784.1	EV215_RS04395	DUF2800 domain-containing protein	PHAGE_Clostr_phiCT453A_NC_028991: hypothetical protein	7.39e-113
2	WP_134112862.1	EV215_RS04400	rRNA biogenesis protein rrp5	rRNA biogenesis protein rrp5	NA
2	WP_134112785.1	EV215_RS04405	hypothetical protein	hypothetical protein	NA
2	WP_134112786.1	EV215_RS04410	LysM peptidoglycan-binding domain-containing protein	PHAGE_Clostr_phiCT453A_NC_028991: dsDNA binding protein	7.36e-07
2	WP_134112787.1	EV215_RS04415	holin	PHAGE_Bacill_PfEFR_5_NC_031055: hypothetical protein	1.02e-31
2	WP_134112863.1	EV215_RS04420	glycosyl hydrolase	PHAGE_Microb_Eleri_NC_042109: hypothetical protein	1.64e-45

2	WP_134112788.1	EV215_RS04425	hypothetical protein	hypothetical protein	NA
2	WP_134112789.1	EV215_RS04430	hypothetical protein	PHAGE_Geobac_TP_84_NC_041918: DCTP pyrophosphatase	2.06e-12
2	WP_134112790.1	EV215_RS04435	hypothetical protein	PHAGE_Geobac_TP_84_NC_041918: hypothetical protein	1.42e-85
2 & 3	WP_134112791.1	EV215_RS04440	phage tail family protein	PHAGE_Clostr_phiCD111_NC_028905: Tail component protein	8.72e-12
2 & 3	Pseudogene	EV215_RS04445	phage tail protein	phage tail protein	NA
2 & 3	WP_134112864.1	EV215_RS04450	hypothetical protein	hypothetical protein	NA
2 & 3	WP_134112792.1	EV215_RS04455	hypothetical protein	hypothetical protein	NA
2 & 3	WP_134112793.1	EV215_RS04460	phage tail protein	PHAGE_Paenib_Harrison_NC_028746: major tail protein	3.31e-31
2 & 3	WP_134112794.1	EV215_RS04465	hypothetical protein	hypothetical protein	NA
2 & 3	WP_134112795.1	EV215_RS04470	HK97 gp10 family phage protein	PHAGE_Bacill_phIS3501_NC_019502: gp10 family phage protein	1.45e-08
2 & 3	WP_134112796.1	EV215_RS04475	head-tail adaptor protein	head-tail adaptor protein	NA
2 & 3	WP_134112797.1	EV215_RS04480	phage gp6-like head-tail connector protein	phage gp6-like head-tail connector protein	NA
2 & 3	WP_134112798.1	EV215_RS04485	Head fiber protein	PHAGE_Escher_Av_05_NC_025830: putative head fiber protein	1.46e-09
2 & 3	WP_134112799.1	EV215_RS04490	phage major capsid protein	PHAGE_Azospi_Cd_NC_010355: Phage major capsid protein	1.67e-45
2 & 3	WP_134112800.1	EV215_RS04495	phage portal protein	PHAGE_Burkho_phi6442_NC_009235: gp4, phage portal protein, HK97 family	9.04e-81
2 & 3	WP_134112801.1	EV215_RS04500	terminase large subunit	PHAGE_Nocard_NBR1_NC_016569: terminase large subunit	2.50e-98
2 & 3	Pseudogene	EV215_RS04505	hypothetical protein	hypothetical protein	NA
2 & 3	WP_134112802.1	EV215_RS04510	DUF5049 domain-containing protein	PHAGE_Halovi_HCTV_1_NC_021330: hypothetical protein	2.61e-09
2 & 3	WP_134112803.1	EV215_RS04515	DUF4314 domain-containing protein	PHAGE_Bacill_SP_15_NC_031245: hypothetical protein	2.90e-07
3	WP_134112804.1	EV215_RS04520	virulence protein	virulence protein	NA
3	WP_134112865.1	EV215_RS04525	DNA (cytosine-5-)-methyltransferase	PHAGE_Salmon_SJ46_NC_031129: hypothetical protein	1.95e-36
3	WP_134112805.1	EV215_RS04530	DNA modification methylase	PHAGE_Strept_EJ_1_NC_005294: transferase	5.56e-74
3	WP_134112806.1	EV215_RS04535	terminase	terminase	NA
3	WP_134112866.1	EV215_RS04540	HNH endonuclease	PHAGE_Bacill_phIS3501_NC_019502: HNH endonuclease domain protein	3.53e-14
3	WP_134112807.1	EV215_RS04545	DUF1492 domain-containing protein	PHAGE_Strept_phiARI0462_NC_031942: DCTP pyrophosphatase	5.52e-07
3	WP_134112808.1	EV215_RS04550	hypothetical protein	hypothetical protein	NA
3	WP_134112809.1	EV215_RS04555	DEAD/DEAH box helicase	PHAGE_Entero_phiFL4A_NC_013644: DNA helicase	3.35e-174
3	WP_134112810.1	EV215_RS04560	VRR-NUC domain-containing protein	PHAGE_Endosy_APSE_1_NC_000935: hypothetical protein	2.30e-20
3	WP_134112811.1	EV215_RS04565	hypothetical protein	PHAGE_Clostr_phiCT453A_NC_028991: hypothetical protein	0.0
3	WP_134112867.1	EV215_RS04570	DUF4406 domain-containing protein	PHAGE_Gordon_Gsput1_NC_030932: ribonucleotide reductase A subunit	6.96e-09
3	WP_134112812.1	EV215_RS04575	hypothetical protein	hypothetical protein	NA
3	WP_134112813.1	EV215_RS04580	transcriptional regulator	PHAGE_Entero_mEp460_NC_019716: regulatory protein Rha	1.01e-21

Supplementary Table 2: HHpred-suggested annotations of prophage-associated genes. GenBank accession numbers represent the corresponding proteins from each locus. Pseudogene entries are noted instead of an accession number. E-values correspond to HHpred annotation for each entry. Non-available E-Values marked as "NA". Entry marked with an asterisk (*) is not found in the three defined regions as it was not annotated by PHASTER (ND), but was manually added to the analysis.

Region	GenBank	Locus tag	HHpred annotation	Prob.	E-Value
1	WP_134112568.1	EV215_RS03310	4LGY_A Phosphoribosylformylglycinamidine synthase (E.C.6.3.5.3)	100	3.00E-138
1	WP_134112569.1	EV215_RS03315	1O4V_A phosphoribosylaminoimidazole mutase PurE (E.C.4.1.1.21)	100	5.8E-29
1	WP_134112570.1	EV215_RS03320	3AX6_A Phosphoribosylaminoimidazole carboxylase, ATPase subunit (E.C.6.3.4.18)	100	1.2E-44
1	WP_134112631.1	EV215_RS03325	2GQR_A Phosphoribosylaminoimidazole-succinocarboxamide synthase (E.C.6.3.2.6)	100	3.9E-62
1	WP_134112571.1	EV215_RS03330	6LBP_A Amidophosphoribosyltransferase 2, chloroplastic (E.C.2.4.2.14)	100	2.9E-63
1	WP_134112572.1	EV215_RS03335	5VK4_A Phosphoribosylformylglycinamidine cyclo-ligase (E.C.6.3.3.1)	100	6.9E-44
1	WP_134112573.1	EV215_RS03340	1JKX_B Phosphoribosylglycinamide formyltransferase (E.C.2.1.2.2)	100	4.9E-25
1	WP_134112574.1	EV215_RS03345	4EHI_B Bifunctional purine biosynthesis protein PurH	100	1.00E-133
ND*	WP_134112775.1	EV215_RS04350	6JIV_B SspE protein; DNase*	100	5.00E-37
2	WP_134112776.1	EV215_RS04355	P13340 END7_BPT4 Recombination endonuclease VII	98.1	4.50E-06
2	WP_134112777.1	EV215_RS04360	hypothetical protein		NA
2	WP_134112778.1	EV215_RS04365	4BQQ_B Integrase	100	4.10E-42
2	WP_134112779.1	EV215_RS04370	5UDO_F Putative integrase	99.7	1.50E-16
2	WP_134112780.1	EV215_RS04375	4BQQ_B Integrase	100	8.20E-43
2	WP_134112781.1	EV215_RS04380	hypothetical protein		NA
2	WP_134112782.1	EV215_RS04385	P06225 DPOL_BPSP2 DNA polymerase OS=*Bacillus* phage SP02	100	4.90E-78
2	WP_134112783.1	EV215_RS04390	4JG2_A Phage-related protein	100	6.10E-37
2	WP_134112784.1	EV215_RS04395	Q9T1P7 VP51_BPAPS Putative protein p51 OS=*Acyrthosiphon pisum* secondary endosymbiont phage 1	100	1.90E-50
2	WP_134112862.1	EV215_RS04400	Q9T1P5 VP53_BPAPS Putative protein p53 OS=*Acyrthosiphon pisum* secondary endosymbiont phage 1	99.5	2.70E-13
2	WP_134112785.1	EV215_RS04405	hypothetical protein		NA
2	WP_134112786.1	EV215_RS04410	4KNK_B Bifunctional autolysin (E.C.3.5.1.28, 3.2.1.96); peptidoglycan, autolysin, amidase, N-acetylmuramoyl-L-alanine amidase	99.7	2.80E-16
2	WP_134112787.1	EV215_RS04415	Q9ZXD8 VLYS_BPPH1 Probable holin	100	4.90E-26
2	WP_134112863.1	EV215_RS04420	3W4R_A Chitinase (E.C.3.2.1.14); insect, glycosyl hydrolase	100	9.90E-30
2	WP_134112788.1	EV215_RS04425	hypothetical protein		NA
2	WP_134112789.1	EV215_RS04430	hypothetical protein		NA
2	WP_134112790.1	EV215_RS04435	Q0PDK6 Fiber_BPSPP Tail spike protein OS=*Bacillus* phage SPP1	100	1.60E-25

2 & 3	WP_134112791.1	EV215_RS04440	2X8K_C Hypothetical protein 19.1; viral protein, distal tail protein	100	6.90E-31
2 & 3	Pseudogene	EV215_RS04445	phage tail protein		NA
2 & 3	WP_134112864.1	EV215_RS04450	PF09550.11 ; Phage_TAC_6 ; Phage tail assembly chaperone protein,	98.5	2.90E-07
2 & 3	WP_134112792.1	EV215_RS04455	PF12363.9 ; Phage_TAC_12 ; Phage tail assembly chaperone protein, TAC	99.4	4.30E-12
2 & 3	WP_134112793.1	EV215_RS04460	PF04630.13 ; Phage_TTP_1 ; Phage tail tube protein	100	1.20E-32
2 & 3	WP_134112794.1	EV215_RS04465	6TE9_F Adaptor protein Rcc01688, Portal protein; "neck", "portal", "capsid", "tail tube"; 3.58A {*Rhodobacter capsulatus*}	99.4	3.30E-11
2 & 3	WP_134112795.1	EV215_RS04470	Q01261 GPG_BPMU Putative capsid assembly protein G OS=Escherichia phage	99.5	2.20E-12
2 & 3	WP_134112796.1	EV215_RS04475	6TE9_E Adaptor protein Rcc01688, Portal protein; "neck", "portal", "capsid", "tail tube"; 3.58A {*Rhodobacter capsulatus*}	99.9	8.80E-22
2 & 3	WP_134112797.1	EV215_RS04480	cd08051 gp6_gp15_like; Head-Tail Connector Proteins gp6 and gp15	99.7	3.40E-15
2 & 3	WP_134112798.1	EV215_RS04485	>P11186 VG85_BPNF Head fiber protein OS=Bacillus phage Nf	99.6	1.80E-15
2 & 3	WP_134112799.1	EV215_RS04490	3QPR_F Major capsid protein; Virus Procapsid particles, VIRUS; 5.2A {Enterobacteria phage HK97}	100	2.00E-37
2 & 3	WP_134112800.1	EV215_RS04495	P49859 PORTL_BPHK7 Portal protein OS=Enterobacteria phage HK97	100	6.80E-32
2 & 3	WP_134112801.1	EV215_RS04500	P59217 TERL_BPSF5 Putative terminase large subunit OS=*Shigella* phage SfV	100	2.00E-47
2 & 3	Pseudogene	EV215_RS04505	hypothetical protein		NA
2 & 3	WP_134112802.1	EV215_RS04510	PF16468.6; DUF5049 ; Domain of unknown function (DUF5049)	100	5.80E-31
2 & 3	WP_134112803.1	EV215_RS04515	PF14192.7; DUF4314 ; Domain of unknown function (DUF4314)	100	7.60E-27
3	WP_134112804.1	EV215_RS04520	PF12224.9; Amidoligase_2 ; Putative amidoligase enzyme	99.9	1.70E-21
3	WP_134112865.1	EV215_RS04525	P09915 MTBR_BPRH1 Modification methylase Rho11sI OS=Bacillus phage	100	7.60E-41
3	WP_134112805.1	EV215_RS04530	1BOO_A protein (N-4 cyotisine-specific methyltransferase PVU; Type II DNA-(cytosine N4) METHYLTRANSFERASE	100	1.30E-26
3	WP_134112806.1	EV215_RS04535	PF05119.13; Terminase_4 ; Phage terminase, small subunit	99.7	1.20E-15
3	WP_134112866.1	EV215_RS04540	5H0M_A HNH endonuclease; Thermophilic bacteriophage, HNH Endonuclease	99.6	4.80E-15
3	WP_134112807.1	EV215_RS04545	O34055 ARPU_BPO12 Putative autolysin regulatory protein ArpU OS=*Streptococcus phage* O1205 OX=85154 GN=arpU	99.2	1.10E-09
3	WP_134112808.1	EV215_RS04550	hypothetical protein		NA
3	WP_134112809.1	EV215_RS04555	Q9T1Q7 VP41_BPAPS Putative protein p41 OS=*Acyrthosiphon pisum* secondary endosymbiont phage 1	100	1.60E-50

3	WP_134112810.1	EV215_RS04560	4QBN_B Uncharacterized protein; Nuclease, hydrolase; HET: SO4; 1.85A {*Salmonella* phage SETP3} SCOP: c.52.1.35;	99.9	2.30E-24
3	WP_134112811.1	EV215_RS04565	P10277 Prim_BPP4 Putative P4-specific DNA primase OS=Enterobacteria phage P4	99.8	2.00E-19
3	WP_134112867.1	EV215_RS04570	1S2L_C purine trans deoxyribosylase (E.C.2.4.2.6); PTD, native, 2'-purine deoxyribosyltansferase	99.6	1.60E-13
3	WP_134112812.1	EV215_RS04575	hypothetical protein		NA
3	WP_134112813.1	EV215_RS04580	P21417 Y23K_BPP22 Uncharacterized protein	98.3	1.00E-04

Supplementary Table 3: Highest identities of HTH1 proteins (EV215_RS03310 to EV215_RS03345) with at least 3 protein hits in Aclame [1] (A CLAssification of Mobile genetic Elements) from the head-neck-tail module analysis performed using VIRFAM [2].

Protein Superfamily	Corresponding Protein in Aclame	Aclame Phage	Seq. Identity (%)
Major Capsid Protein (MCP)	protein:vir:4511	V	29 %
	protein:vir:4456	ST64B	29 %
	protein:vir:485	P27	29 %
Portal	protein:vir:100150	phi1026b	38 %
	protein:vir:80333	phi644-2	36 %
	protein:vir:1431	phiE125	36 %
Terminase (TermL)	protein:vir:4335	D3	34 %
	protein:vir:81178	*Geobacillus* virus E2	33 %
	protein:vir:105086	phiKO2	33 %
Major Tail Protein (MTP)	protein:vir:95370	GBSV1	26 %
	protein:vir:102037	Fah	26 %
	protein:vir:102845	Cherry	26 %
Adaptor (Ad1)	protein:vir:1384	phi3626	28 %
	protein:vir:4512	V	26 %
	protein:vir:1271	phi-105	26 %
Head Closure (Hc1)	protein:vir:81177	*Geobacillus* virus E2	22 %
	protein:vir:1890	HK022	22 %
	protein:vir:193	HK97	21 %
Neck (Ne1)	protein:vir:102085	Fah	26 %
	protein:vir:102875	Cherry	26 %
	protein:vir:107568	Gamma	26 %
Tail Completion (Tc1)	protein:vir:1274	phi-105	26 %
	protein:vir:1387	phi3626	23 %
	protein:vir:102086	Fah	18 %

Supplementary Table 4: Amino acid sequence comparisons of target HTH1 proteins to their homologs in the three chosen viral gene clusters, informed by protein-protein blast (blastp) searches using NCBI BLAST [3]. A Protein Data Bank (PDB) [4] identifier is included of the PDB hit with the highest similarity, where available. NA indicates no match/no data available.

Hypnocyclicus thermotrophus phage H1		IMGVR		NCBI				Protein Data Bank (PDB)		
		Marine anoxygenic community R3		Streptococcus phage Javan630		Erysipelothrix phage phi1605				
Locus Tag	Identifier	% Identity	% Query Coverage	% Identity	% Query Coverage	% Identity	% Query Coverage	% Identity	% Query Cover	ID
EV215_RS04435	HTP4435	98	100	83.23	100	82.76	100	NA	NA	NA
EV215_RS04425	HTP4425	94	100	80.41	100	78.87	100	NA	NA	NA
EV215_RS04420	HTP4420	98	99	80	99	80.61	99	26.34	28	4S3J
EV215_RS04415	HTP4415	99	100	75.74	100	75	100	NA	NA	NA
EV215_RS04410	HTP4410	93	100	NA	NA	NA	NA	34.92	18	3HMB
EV215_RS04400	HTP4400	96	100	55.13	74	65.38	99	NA	NA	NA
EV215_RS04385	HTP4385	89	100	72.51	64	75.91	100	25.64	23	1D9D
EV215_RS04360	HTP4360	97	100	NA	NA	NA	NA	NA	NA	NA
EV215_RS04350	HTP4350	97	100	NA	NA	NA	NA	NA	NA	NA

Supplementary Table 5: Detailed information on the HTH1 proteins chosen for expression trials. Candidate protein molecular weight (Mw) and isoelectric point (pI) calculated using ExPASy compute pI/Mw tool [5]. His6 indicates the hexahistidine affinity tag.

Identifier	Mw (kDa)	pI	Proposed Function	Vector	His-Tag
HTP4435	93	4.73	Endopeptidase tail	pET-21b (+)	C-terminal His6
HTP4425	22	6.83	Hypothetical protein	pET-21b (+)	C-terminal His6
HTP4420	94	4.88	Glycosyl hydrolase 18	pET-21b (+)	C-terminal His6
HTP4415	15	5.05	Holin, toxin secretion/phage lysis	pET-21b (+)	C-terminal His6
HTP4410	35	9.06	N-acetylmuramoyl-L-alanine amidase	pET-21b (+)	C-terminal His6
HTP4400	11.5	5.45	rRNA biogenesis protein rrp5, putative	pET-21b (+)	C-terminal His6
HTP4385	74	8.79	DNA Polymerase	pET-21b (+)	C-terminal His6
HTP4360	20	5.48	hypothetical protein	pET-21b (+)	C-terminal His6
HTP4350	66	5.92	DNase	pET-21b (+)	C-terminal His6

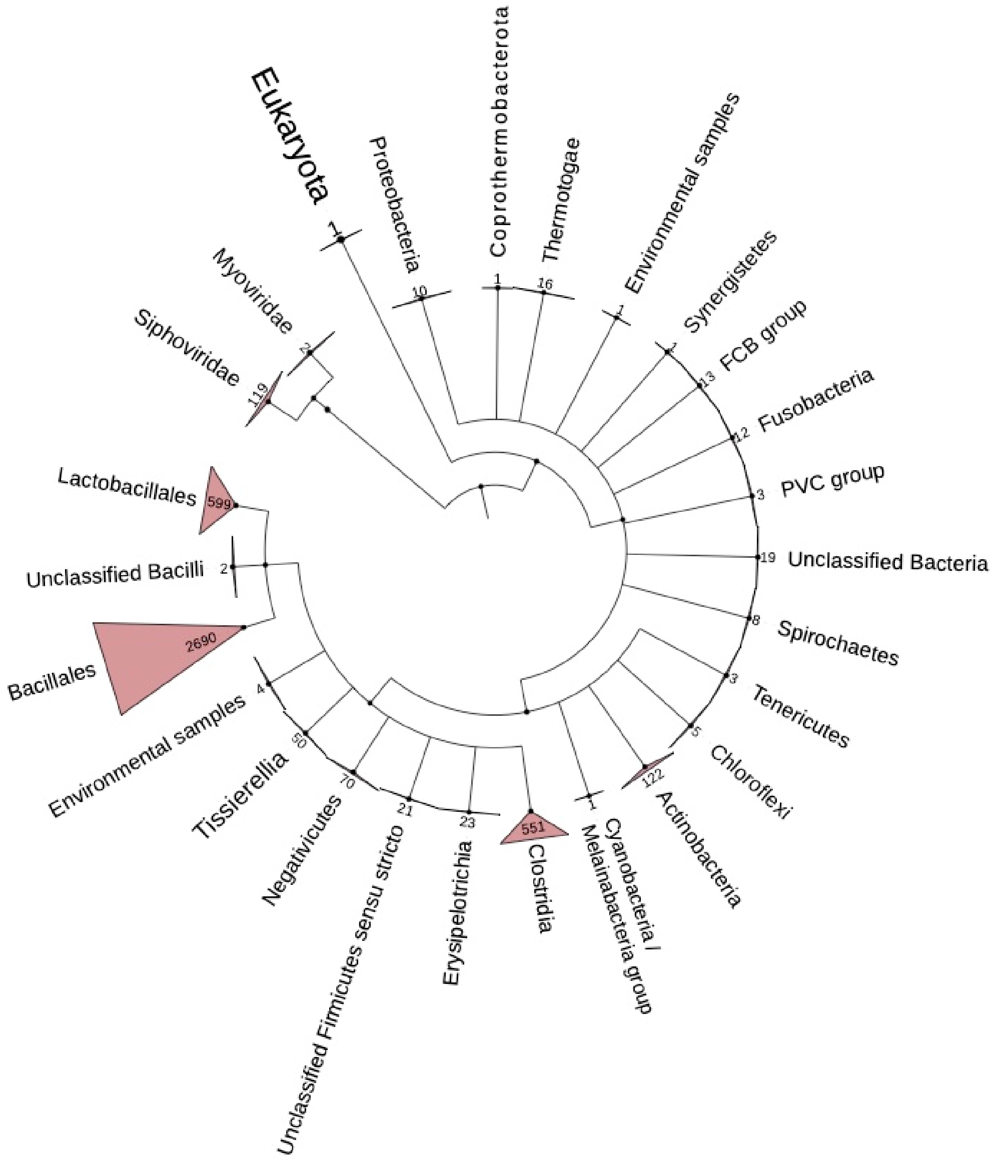

Supplementary Figure 1: Taxonomic species distribution of the blastx search results of the HTH1 genes against the NCBI non-redundant (nr) nucleotide database, generated as described in the materials and methods section. Numbers next to each node indicate the number of unique species represented by each cartoon.

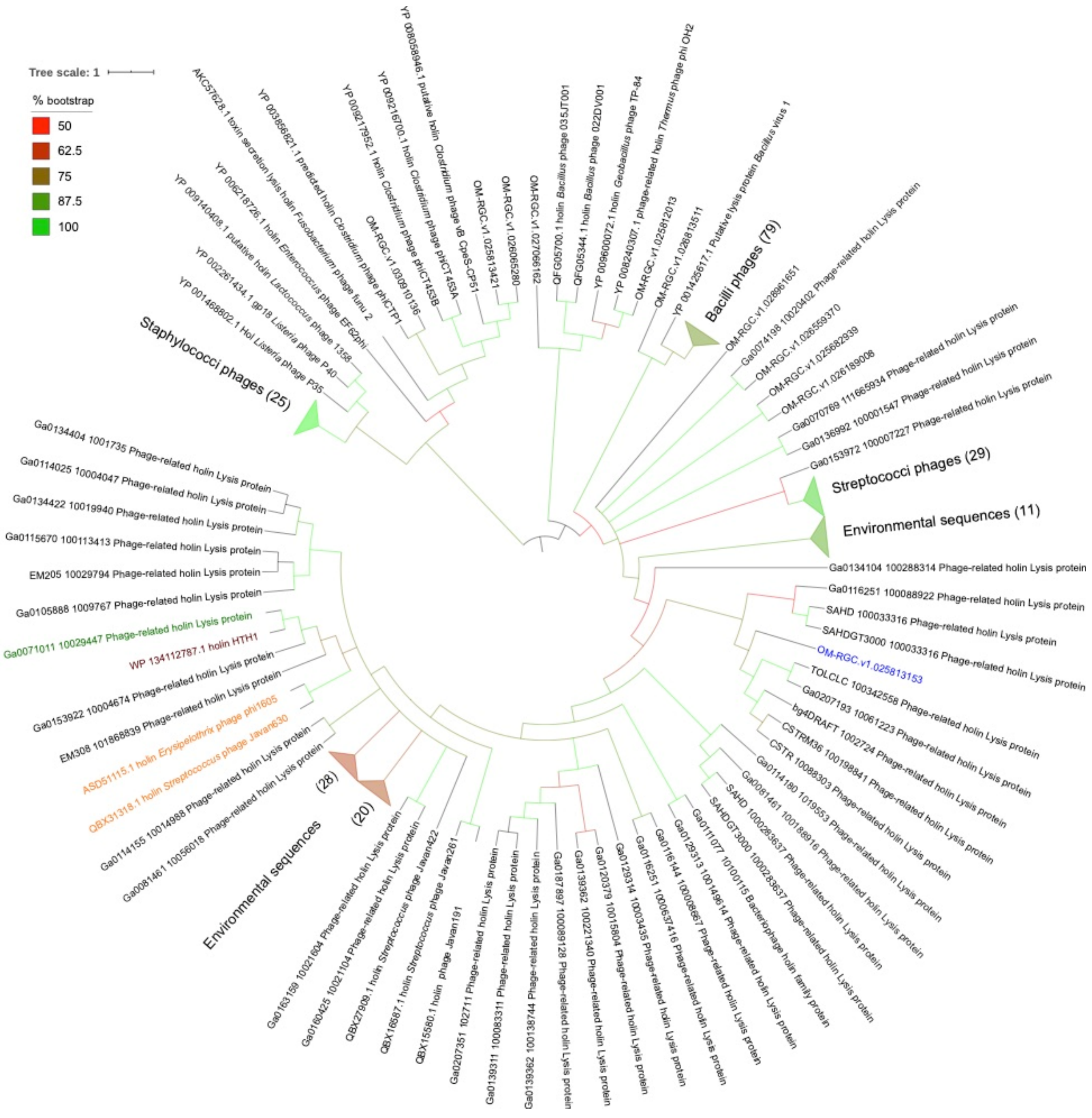

Supplementary Figure 2: Phylogeny analysis of the prophage based on the alignment of 94 amino acid long region of holins from 211 phages and phage-related environmental sequences. The tree was constructed as described within the materials and methods section. Numbers next to collapsed clades represent the number of leaves covered by each cartoon. The HTH1 holin is highlighted in red. The closest environmental entry from the IMG/VR database is highlighted in green and from the Ocean Gene Atlas in blue. The closest entries from known phages described on NCBI are highlighted in orange. The only other Fusobacterium phage-related entry FUNU2 is shown in bold.

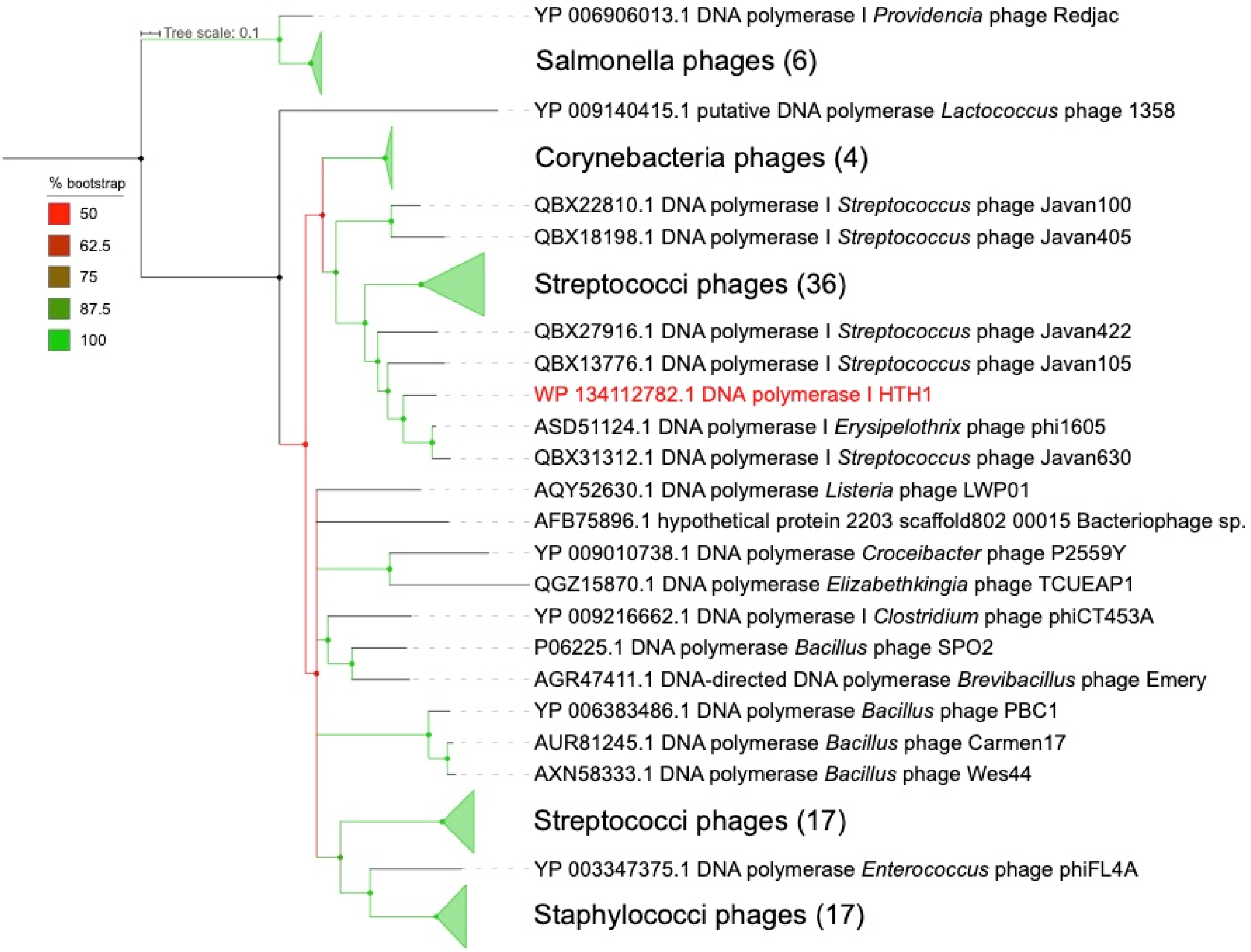

Supplementary Figure 2: Phylogeny analysis of 625 amino acids long alignment of the DNA polymerase I proteins from 101 top phage hits using maximum likelihood with 1000 bootstrap replicates. The tree is center-rooted and the scale bar represents the average number of amino acid substitutions per site. Numbers next to collapsed clades represent the number of leaves covered by each cartoon. The HTH1 entry is highlighted in red.

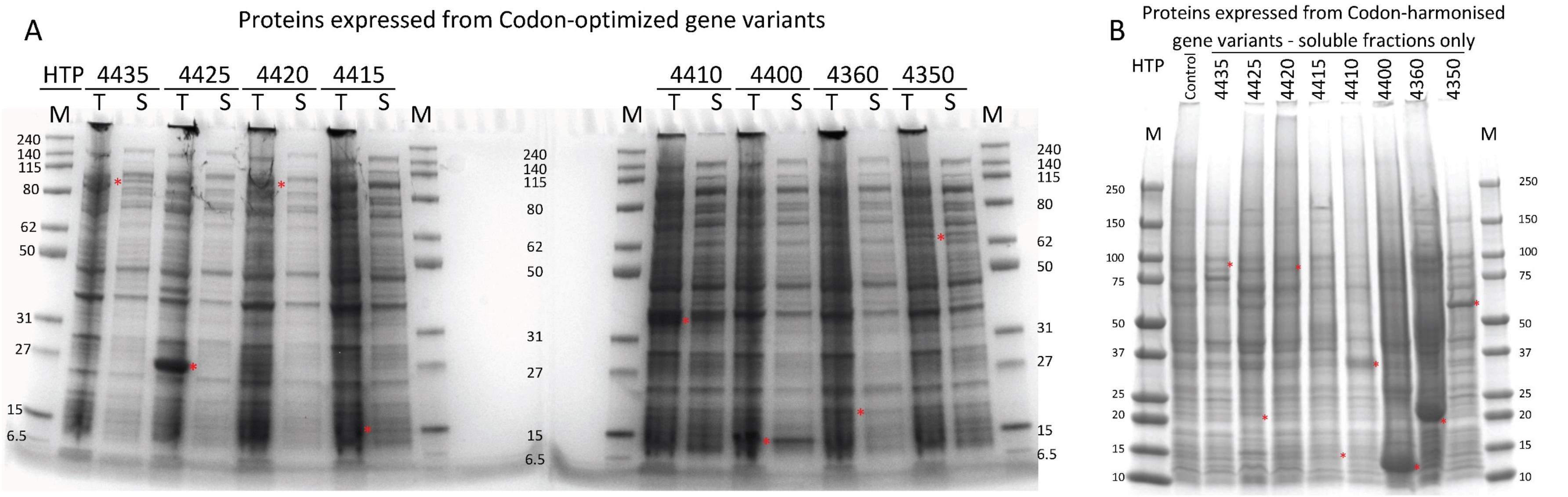

Supplementary Figure 3: SDS-PAGE images from protein (over)expression trials with (A) Codon-harmonized and (B) Codon-optimized sets of genes. The HTP prefix and the numbers above the lanes correspond to the identifiers of the genes tested. T and S refer to total (crude) and soluble (clear) lysate fractions, respectively. M indicates the protein marker. Numbers next to each protein marker lane show the respective molecular weight labels, in kDa. Red asterisks indicate the expected position of each protein, inferred from their molecular weight.

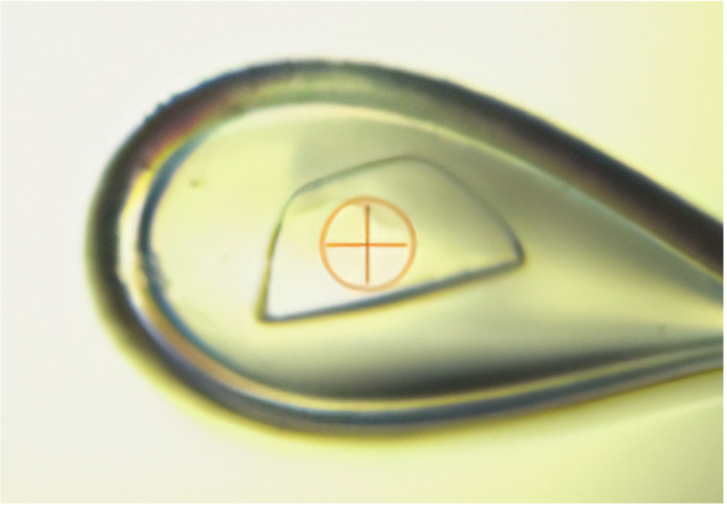

Supplementary Figure 5: Protein crystal of HTP4350 produced from codon-harmonized gene variant pictured within the loop, mounted for diffraction data collection.

References:

1. Leplae, R.; Lima-Mendez, G.; Toussaint, A. ACLAME: A CLAssification of Mobile genetic Elements, update 2010. *Nucleic Acids Res.* **2010**, *38*, D57–D61, doi:10.1093/nar/gkp938.
2. Lopes, A.; Tavares, P.; Petit, M.A.; Guérois, R.; Zinn-Justin, S. Automated classification of tailed bacteriophages according to their neck organization. *BMC Genomics* **2014**, *15*, doi:10.1186/1471-2164-15-1027.
3. Altschul, S.F.; Gish, W.; Miller, W.; Myers, E.W.; Lipman, D.J. Basic local alignment search tool. *J. Mol. Biol.* **1990**, *215*, 403–410, doi:10.1016/s0022-2836(05)80360-2.
4. Berman, H.M.; Westbrook, J.; Feng, Z.; Gilliland, G.; Bhat, T.N.; Weissig, H.; Shindyalov, I.N.; Bourne, P.E. The Protein Data Bank. *Nucleic Acids Res.* **2000**, *28*, 235–242, doi:10.1093/nar/28.1.235.
5. Swiss Institute of Bioinformatics ExPASy - Compute pI/Mw tool. Available online: https://web.expasy.org/compute_pi/ (accessed on Apr 14, 2018).
