# Supplementary material for: Exploring Codon Adjustment Strategies towards Escherichia coli-Based Production of Viral Proteins Encoded by HTH1, a Novel Prophage of the Marine Bacterium Hypnocyclicus thermotrophus
Source: Viruses. 2021 Jun 23;13(7):1215. doi: 10.3390/v13071215 (PMC8310279; doi:10.3390/v13071215)
Supplement: Supplementary file 1 [file viruses-13-01215-s001.zip › Supplementary Figure 4 - SDSPage gel images.pdf]

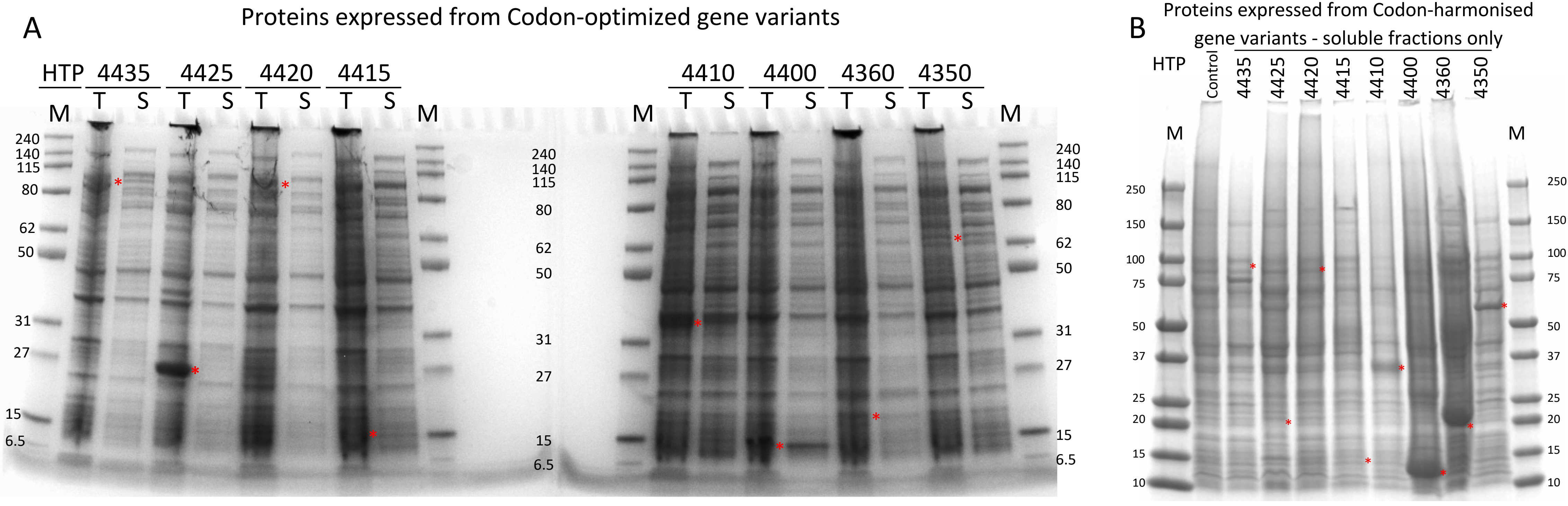

A
Proteins expressed from Codon-optimized gene variants
HTP
4435
4425
4420
4415
4410
4400
4360
4350
T S
M
240
140
115
80
62
50
31
27
15
6.5
B
Proteins expressed from Codon-harmonised gene variants - soluble fractions only
HTP
Control
M
250
150
100
75
50
37
25
20
15
10
