# Supplementary figures and images for: Exploring Codon Adjustment Strategies towards Escherichia coli-Based Production of Viral Proteins Encoded by HTH1, a Novel Prophage of the Marine Bacterium Hypnocyclicus thermotrophus
Source: Viruses. 2021 Jun 23;13(7):1215. doi: 10.3390/v13071215 (PMC8310279; doi:10.3390/v13071215)

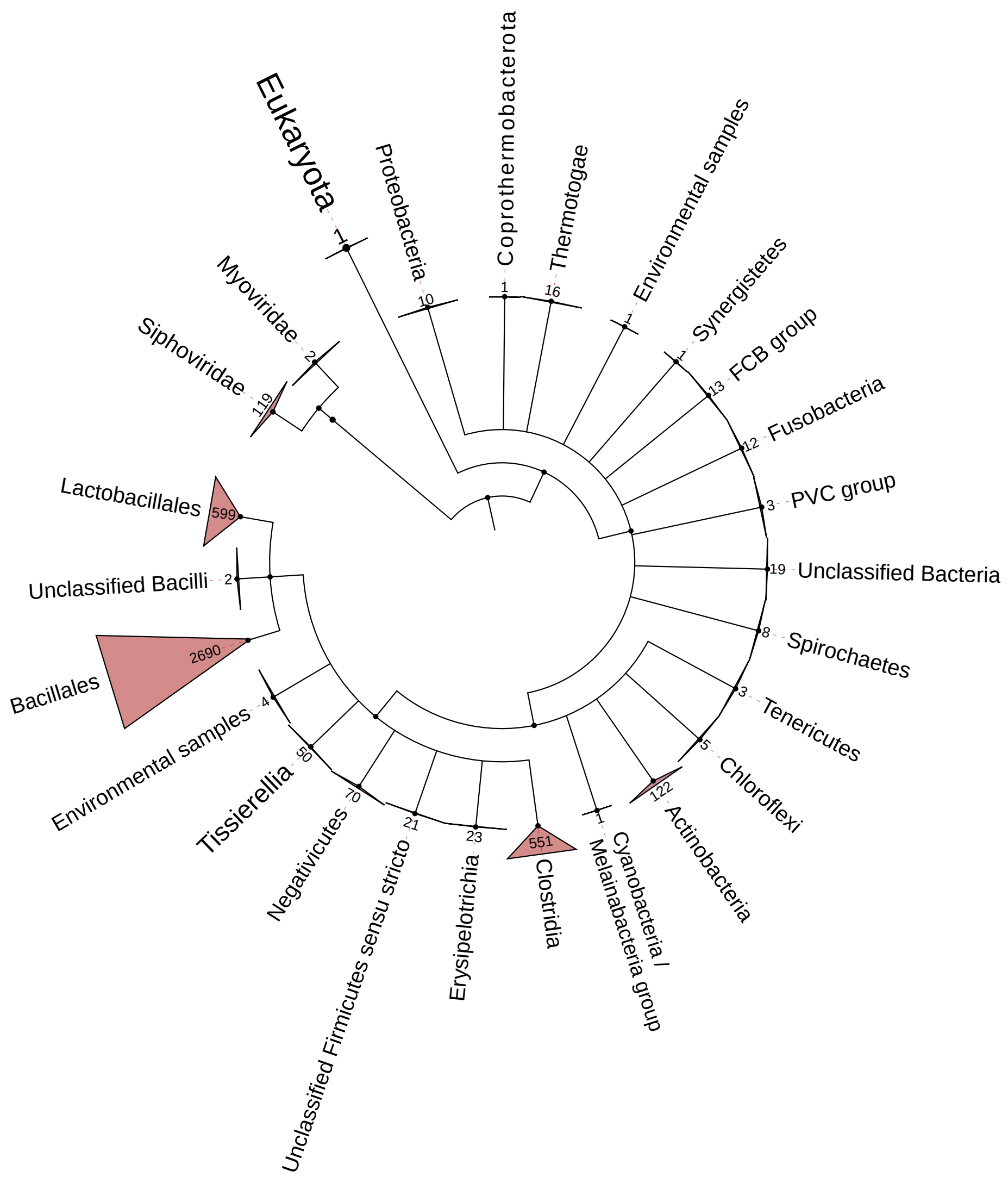

Supplement: Supplementary file 1 [file viruses-13-01215-s001.zip › Supplementary Figure 1 - Taxonomical distribution of phage hosts.pdf]

Tree scale: 1 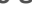

```
% bootstrap
```

50

■ 62.5

75

87.5

100

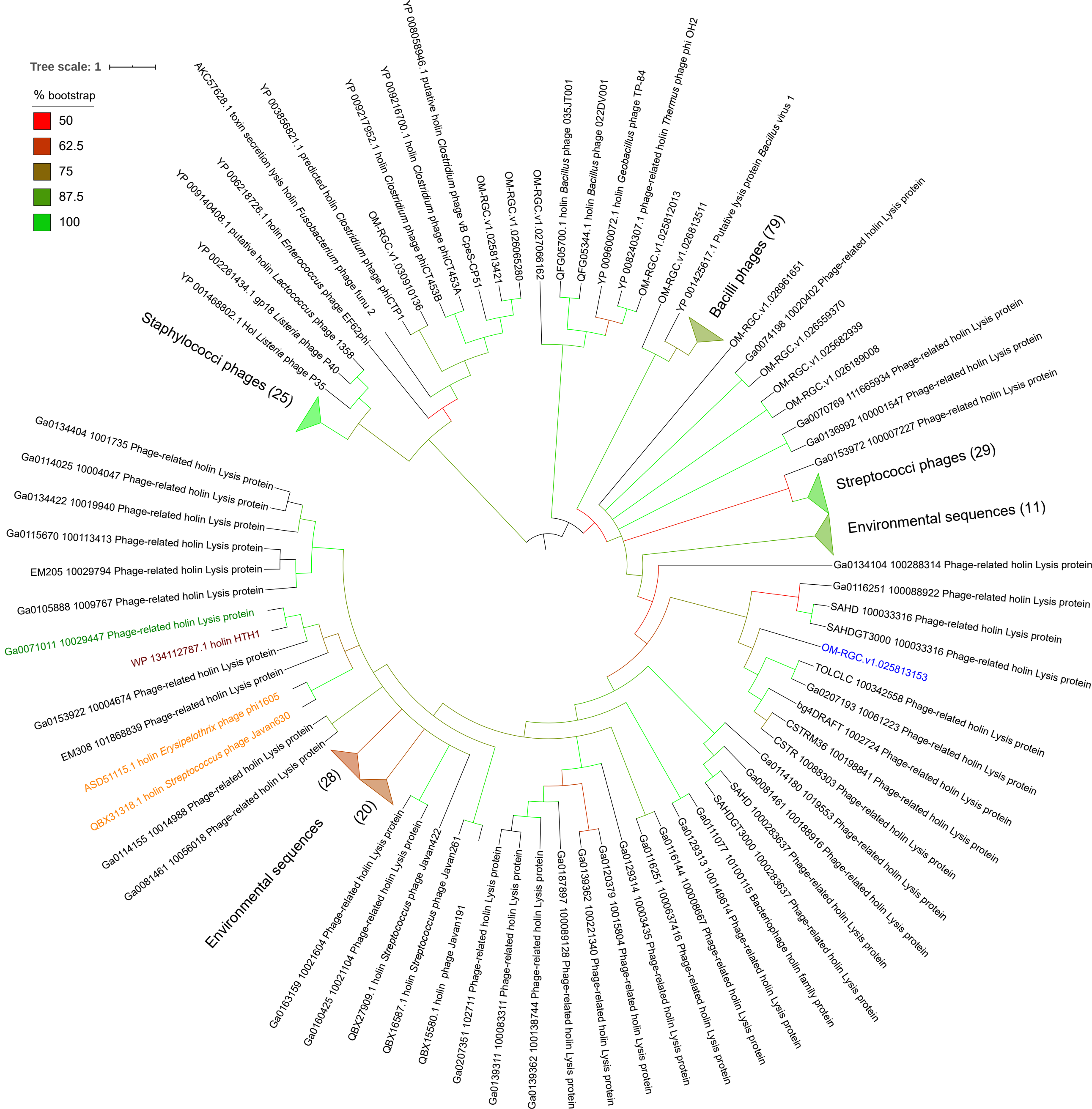

Supplement: Supplementary file 1 [file viruses-13-01215-s001.zip › Supplementary Figure 2 - Extended Holin phylogenetic analysis.pdf]

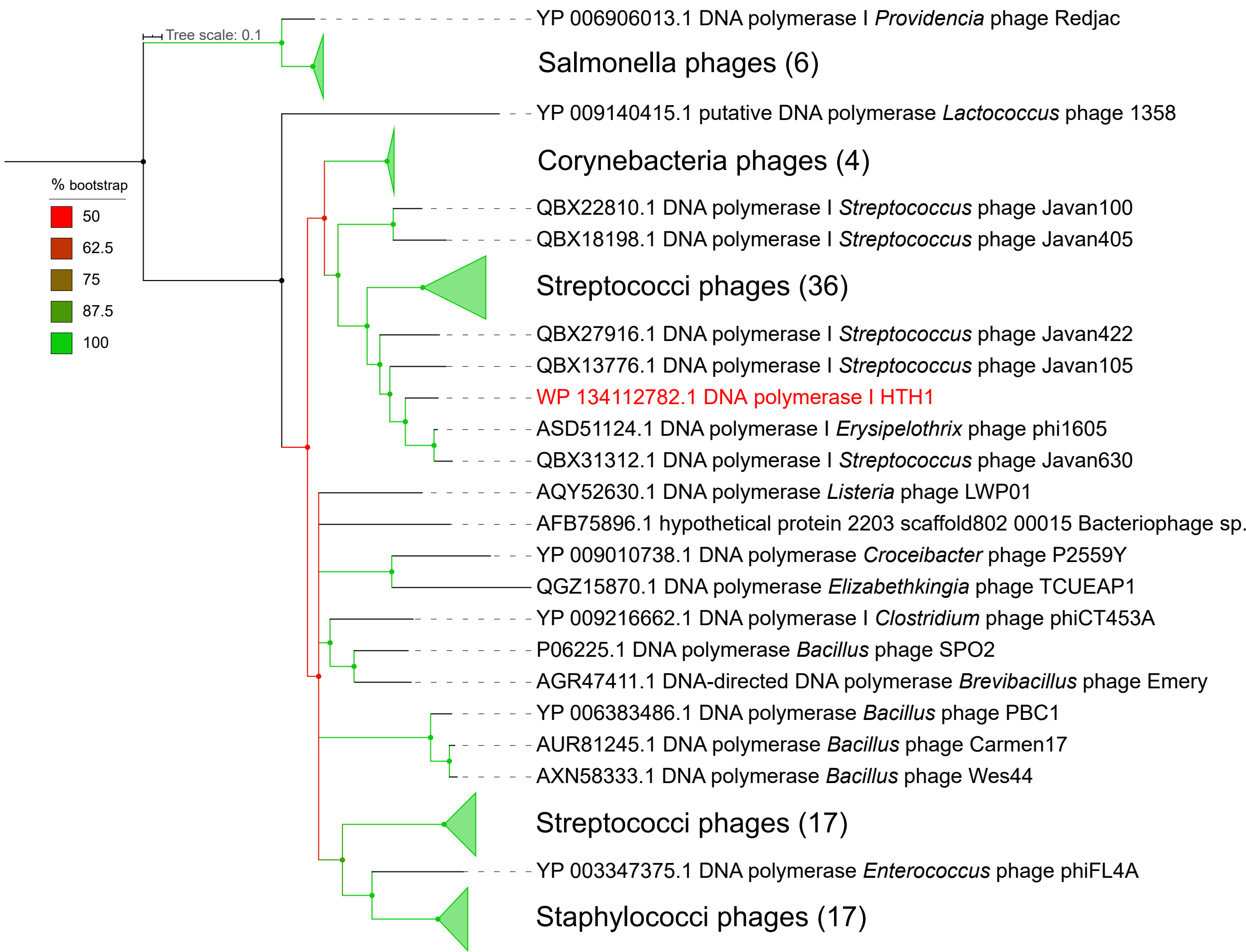

Supplement: Supplementary file 1 [file viruses-13-01215-s001.zip › Supplementary Figure 3 - DNA polymerase phylogenetic analysis.pdf]

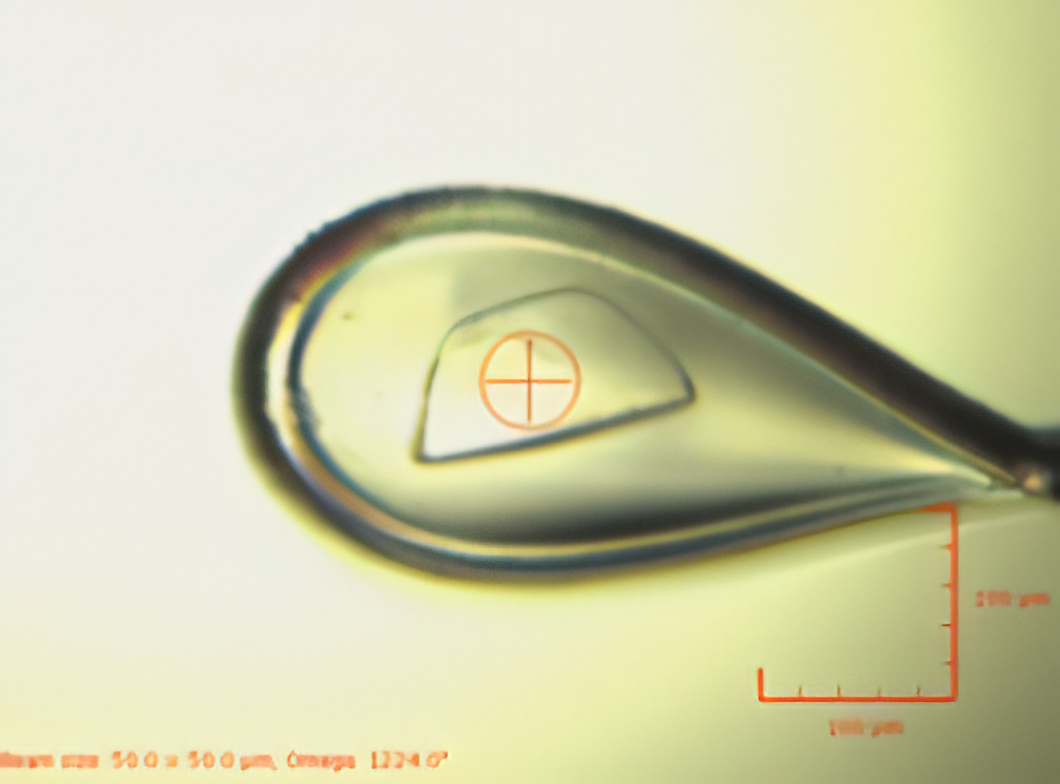

Supplement: Supplementary file 1 [file viruses-13-01215-s001.zip › Supplementary Figure 5 - Crystal Image.png]
